# Comprehensive Profiling of Plasma Fatty Acid Concentrations in Young Healthy Canadian Adults

**DOI:** 10.1371/journal.pone.0116195

**Published:** 2015-02-12

**Authors:** Salma A. Abdelmagid, Shannon E. Clarke, Daiva E. Nielsen, Alaa Badawi, Ahmed El-Sohemy, David M. Mutch, David W. L. Ma

**Affiliations:** 1 Department of Human Health and Nutritional Sciences, University of Guelph, Guelph, Ontario, Canada; 2 Department of Nutritional Sciences, University of Toronto, Toronto, Ontario, Canada; 3 Office for Biotechnology, Genomics and Population Health, Public Health Agency of Canada, Toronto, Ontario, Canada; University of Basque Country, SPAIN

## Abstract

Circulating fatty acids (FA) are associated with a multitude of chronic diseases. However, a major gap in establishing such relationships is the lack of accepted fatty acid reference ranges representing healthy individuals. Data on validated FA reference ranges would provide a better understanding of study baseline measures and aid in the evaluation and interpretation of pharmaceutical or dietary interventions. Reference ranges for plasma FA levels have been reported in a few small studies and on a limited number of FA. Therefore, we determined the average and percentiles of a broad set of 61 FA (C14 - C24:1) from plasma total lipids from an ethnically diverse population of healthy young Canadian males and females (Total n = 826). Plasma concentrations of some of the major FA ranged from 0.3 to 4.1 mmol/L for palmitic acid, 0.1 to 1.0 mmol/L for stearic acid, 0.03 to 3.2 mmol/L for oleic acid, 0.2 to 5.0 mmol/L for linoleic acid (LA), 12.0 to 186.9 μmol/L for α-linolenic acid, and 7.2 to 237.5 μmol/L for docosahexaenoic acid (DHA). Males had significantly higher plasma concentrations of γ-linolenic acid (GLA) and n-3 docosapentaenoic acid and lower concentrations of palmitoleic acid, LA and DHA than females. Comparison of FA concentrations between Caucasians, East Asians and South Asians revealed that South Asians had significantly lower levels of palmitoleic acid (p < 0.01) and oleic acid (p = 0.01) while East Asians had lower levels of GLA (p = 0.02) and dihomo-γ-linolenic acid (p = 0.03). Overall, these data provide a comprehensive set of quantitative values that profiles a small cohort of Canadians which highlights the utility of establishing validated FA reference ranges that may be used to understand how deficient, suboptimal, or excess amounts of a given FA may be associated with chronic disease.

## Introduction

Currently, there is a fundamental gap in the field of fatty acids (FA) research that hinders the translation and utilization of current knowledge into clinical practice for the prevention and management of chronic diseases. A large body of work has made evident the important influence of dietary and circulating FA in health and disease. FA are implicated in chronic diseases such as cardiovascular and heart disease, cancer, inflammation and autoimmune disease [1–7]; however, despite their recognized ability to modify the risk of disease, “normal” levels of circulating fatty acids are yet to be defined. The lack of established reference ranges for saturated, trans, monounsaturated and polyunsaturated FA has resulted in the poor interpretability of human research [8]. Clinical reference values, obtained by objective clinical measures and not estimated from dietary assessment, are established for many types of lipids including LDL-cholesterol, HDL-cholesterol, total cholesterol, triglycerides, and total free fatty acids. FA linked to these lipids are just as important in relation to short and long term health. As such, a recent study reported associations between serum FA and certain types of ischemic strokes [9]. Clinical reference ranges of FA will allow definitive identification of deficiencies or excesses associated with poor health and would make it possible to establish healthy targets. Yet, the identification of such abnormalities requires first knowledge of the normal distribution of individual circulating FA concentrations. Thus, measurement of FA concentrations in young healthy adults will provide a distribution of values from which identification of age- and disease-related changes is attainable. In that regard, we sought to determine the average concentrations (μmol/L) of 61 FA in total plasma of young healthy Canadians in a cross-sectional study.

## Subjects and Methods

### Study population

Participants were recruited as part of the cross-sectional Toronto Nutrigenomics and Health (TNH) Study [10] between September 2004 and July 2009. Participants ethno-cultural groups were self reported and these included Caucasian, Asian, African, South Asian, Middle eastern, Hispanic, Native American and Jewish. Ages of participants ranged between 20 and 29 years old and written informed consent was obtained from all of those who participated. Participants were a random sample of free living subjects consuming their usual diet. Anthropometric measurements were recorded for all participants and health, lifestyle, and food frequency questionnaires were completed by subjects. Standard clinical procedure was followed for the measurement of glucose, insulin, total-, LDL- and HDL-cholesterol, triglycerides, and free fatty acids [10] (S1 Table). HOMA-IR was calculated using the homeostasis model assessment method [11] (S1 Table). Total energy intake from fat and physical activity scores were calculated from completed questionnaires as previously described [10,12] (S1 Table). Women who were pregnant or breastfeeding were not included in the study. Exclusion criteria for the analysis consisted of: smoking, underlying health problems and use of hormonal contraceptives. The study protocol was approved by the Research Ethics Boards at the University of Toronto and University of Guelph.

### Gas chromatography analysis

Subjects were required to fast overnight for a minimum of 12 h prior to blood collection, separation of plasma and subsequent freezing of samples at -80°C. Frozen plasma samples were thawed on ice for 30 min and a mixture of chloroform: methanol (2:1 v/v) was added to a 50 μl aliquot and analyzed as described previously [13]. In brief, free fatty acid C17:0 was used as an internal standard (5 μg of 1 mg/ml stock). Samples were flushed with nitrogen gas prior to storage over night at 4°C. The next day, samples were subjected to a double extraction. The lower organic phase containing lipids were dried down under a gentle stream nitrogen then saponified by KOH in methanol for 1 hour and subsequently methylated by boron trifluoride (14%) for 1 hour. Fatty acid methyl esters (FAME) were quantified as previously described by gas chromatography [14]. FA peak areas were determined using EZChrom Elite software (Version 3.3.2) [15]. The internal standard was used to calculate FA concentrations (μmol/L) (S1 Table). The responsiveness of the detector was routinely checked against the composition of a commercial mixture of FAME standards.

### Statistical analysis of Data

Results are expressed as mean ± standard deviation (SD). All data was analyzed using JMP genomics software V5 (SAS Institute, Cary, NC). A Tukey’s Honestly Significant Difference test was used to determine significant differences in mean biomarkers of health and FA concentrations between sexes and ethnicities. P values were adjusted for age, BMI, sex/ethnicity, % energy from dietary fat and physical activity in linear regression models. A p-value of ˂ 0.05 was considered statistically significant and Bonferroni correction was used to account for multiple testing.

## Results and Discussion

**Fig 1:**
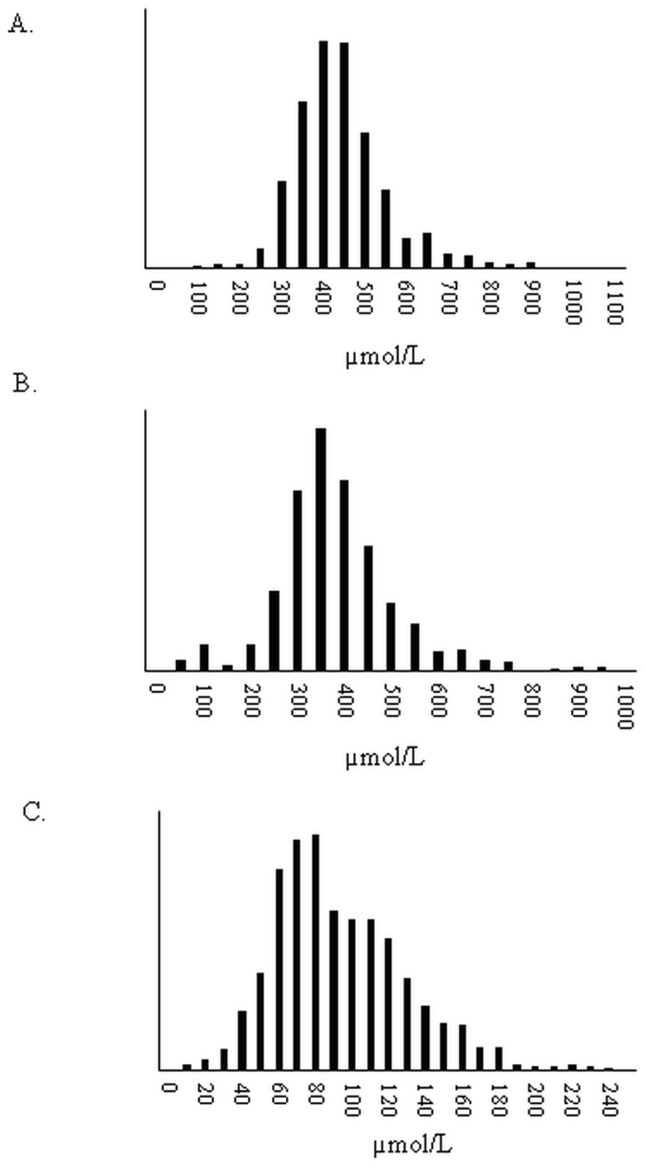
Distribution of total plasma fatty acid concentrations of selected fatty acids. A. 16:0 (Palmitic acid); B. 18:1 c9 (Oleic acid); C. 22:6n-3 (DHA).

In this study we determined, average concentrations of 61 FA in total plasma of young healthy and ethnoculturally-diverse Canadians. We also identified differences in FA concentrations between males and females and between Caucasian, East Asian, and South Asian Canadians. The general characteristics of the study population are presented in Table 1 and are compared by sex and ethnicity in Tables 1 and 2, respectively. Concentrations and percentiles for FA were determined in 826 healthy young individuals (Table 3) and examples of the normal distribution of the wide range of fatty acid concentrations are demonstrated in Fig 1. In 1994, Sera et al determined reference ranges for lauric acid, myristic acid, palmitic acid, palmitoleic acid, stearic acid, oleic acid, linoleic acid, linolenic, homo-γ-linolenic acid and arachidonic acid in American males and females aged 18–55 (n = 128) [16]. Reference range values from these limited FA are similar; however the larger sample size in the present study reveals an even greater range of values. This wider range of concentrations forms a normal distribution as shown in Fig 1. Lower minimums are likely due to the greater sample size in the present study or potentially reflects dietary changes over the past two decades. In contrast with the current study, the vast majority of studies examining circulating FA levels have measured a smaller subset of FA, and reported values of FA as percent composition (summarized in Table 4). The problem of presenting findings in such a manner is the difficulty in comparing results since percent composition values depend on the basket of FA investigated. A study investigating the validity of reporting FA as concentrations compared to weight percentages found that using the latter method of reporting led to the loss of significant differences in FA profiles between groups [17]. Thus, FA concentrations are more useful for facilitating comparisons between studies; hence, we recommend that future studies examining FA levels determine FA concentrations. It is worth noting that studies that have claimed to report FA reference ranges have done so by determining FA concentrations in healthy populations without determining whether these FA are associated with the risk of specific disease [16,18]. Thus, validated reference ranges are yet to be truly established. Also, in establishing high quality reference values it will be important to use multiple internal standards to account for differential responses by FA of different chain length. Thus the inclusion of C17:0 and C21:0 may be appropriate for quantifying long and very long chain FA. Another important consideration when selecting relative or concentrations for establishing references is how these values are used. Studies using concentration values may potentially be more appropriate than relative FA values in association studies with chronic disease biomarkers. Concentrations values are not dependent upon the relative abundance of other FA which is the case when FA are reported as mol% or wt% (area under curve). Reporting of relative FA values is common in nutrition studies and easier to determine than a quantitative approach. Therefore, the association of a relative value to a biomarker is influenced by other FA. Therefore, concentration values which are measured independent of other FA would reflect a direct relationship between what is measured and a given biomarker or outcome.

**Table 1:** General characteristics of study population.

	Total Population	Males	Females	p-value
Population (#)	826	327	499	
Age (yrs)	22.6 ± 2.5	22.8 ± 2.5	22.5 ± 2.5	0.08
BMI (kg/m^2^)	22.8 ± 3.4	23.6 ± 3.3	22.2 ± 3.3	˂ 0.01*
HOMA-IR	1.4 ± 1.3	1.4 ± 1.1	1.4 ± 1.3	0.59
Glucose (mmol/L)	4.8 ± 0.4	4.9 ± 0.4	4.8 ± 0.4	˂ 0.01*
Insulin (pmol/L)	46.2 ± 38.2	44.0 ± 30.4	47.6 ± 42.6	0.19
Total cholesterol (mmol/L)	4.2 ± 0.7	4.1 ± 0.7	4.2 ± 0.7	˂ 0.01*
HDL-cholesterol (mmol/L)	1.5 ± 0.4	1.3 ± 0.3	1.6 ± 0.4	˂ 0.01*
LDL-cholesterol (mmol/L)	2.3 ± 0.6	2.3 ± 0.7	2.3 ± 0.6	0.27
Triglycerides (mmol/L)	0.9 ± 0.4	1.0 ± 0.5	0.8 ± 0.3	˂ 0.01*
Free fatty acids (μmol/L)	474.6 ± 251.7	457.5 ± 252.5	485.9 ± 250.8	0.11
% Energy from dietary fat	27.0 ± 6.0	26.8 ± 6.0	27.1 ± 6.1	0.43

Data represented as Mean±SD.

*A p-value < 0.05, determined by Tukey’s HSD for differences between males and females, was considered statistically significant.

**Table 2:** General characteristics of study population compared by ethnicity.

Fatty Acid	Caucasians	East Asians	South Asians	p- value
Population (#)	287	353	107	
Age (yrs)	23.0 ± 2.5	22.1 ± 2.3	22.4 ± 2.5	
BMI (kg/m^2^)	23.5 ± 3.3^a^	21.8 ± 2.6^b^	23.3 ± 3.9^a^	˂ 0.01*
HOMA-IR	1.3 ± 1.5^b^	1.3 ± 1.0^b^	1.9 ± 1.3^a^	˂ 0.01*
Glucose (mmol/L)	4.8 ± 0.4^b^	4.8 ± 0.4^b^	5.0 ± 0.4^a^	˂ 0.01*
Insulin (pmol/L)	42.2 ± 46.2^b^	43.7 ± 28.4^b^	61.7 ± 40.4^a^	˂ 0.01*
Total cholesterol (mmol/L)	4.1 ± 0.7^b^	4.3 ± 0.7^a^	4.1 ± 0.8^a, b^	0.03*
HDL-cholesterol (mmol/L)	1.5 ± 0.4^b^	1.6 ± 0.4^a^	1.3 ± 0.3^c^	˂ 0.01*
LDL-cholesterol (mmol/L)	2.2 ± 0.6^b^	2.3 ± 0.6^a, b^	2.4 ± 0.7^a^	0.04*
Triglycerides (mmol/L)	0.9 ± 0.4	0.9 ± 0.4	0.9 ± 0.4	0.29
Free fatty acids (μmol/L)	464.2 ± 253.8^a, b^	503.4 ± 265.0^a^	420.5 ± 214.8^b^	0.02*
% Energy from dietary fat	27.8 ± 6.6^a^	26.3 ± 5.1^b^	26.6 ± 7.3^a, b^	˂ 0.01*

Data represented as Mean±SD. A p-value < 0.05, determined by Tukey’s HSD, was considered statistically significant. Different letters (a/b) denote values that are significantly different between groups.

* denote p-values that are significant.

**Table 3:** Range, mean and percentiles of FA concentrations (μmol/L) of plasma total lipids.

Fatty Acid	Min	Mean±SD	Max	10	25	50	75	90
14:0 (Myristic acid)	16.2	63.6 ± 37.1	325.7	29.8	39.2	54.0	76.2	104.7
15:0 (Pentadyclic acid)	*t*	17.8 ± 6.7	56.1	10.8	13.2	17.1	21.3	26.3
16:0 (Palmitic acid)	285.4	1631.1 ± 459.3	4064.5	1140.2	1339.7	1569.5	1839.2	2182.2
18:0 (Stearic acid)	110.2	489.5 ± 124.3	1013.7	353.3	406.9	474.2	556.8	650.9
19:0	*t*	4.3 ± 4.2	25.7	*t*	1.1	3.1	6.6	10.2
20:0 (Arachidic acid)	*t*	4.9 ± 3.7	29.8	*t*	2.6	4.6	6.4	9.4
21:0	*t*	1.5 ± 1.9	10.0	*t*	*T*	0.3	2.9	4.1
22:0 (Behenic acid)	*t*	6.7 ± 5.3	39.0	*t*	3.4	6.0	9.4	14.0
24:0 (Lignoceric acid)	*t*	1.4 ± 2.4	15.7	*t*	*T*	*t*	2.4	5.1
								
14:1 (Myristoleic acid)	*t*	2.7 ± 4.1	31.3	*t*	*T*	*t*	4.2	7.7
15:1 c10	*t*	0.1 ± 0.3	2.7	*t*	*T*	*t*	*t*	*t*
16:1 c9 (Palmitoleic acid)	27.7	133.0 ± 67.2	555.9	67.8	88.5	119.5	156.9	211.4
17:1 c10	*t*	10.5 ± 7.4	45.2	*t*	6.2	10.7	14.7	19.1
18:1 c9 (Oleic acid)	178.7	1285.5± 416.7	3210.5	858.6	1007.1	1226.3	1472.0	1808.7
18:1 c11 (Vaccenic acid)	11.4	129.2 ± 59.5	562.9	81.5	96.6	114.3	141.5	185.1
18:1 c12	*t*	18.7 ± 13.6	101.8	6.6	9.0	14.9	22.7	38.3
18:1 c13	*t*	3.5 ± 3.5	18.1	*t*	1.2	2.5	4.5	8.9
18:1 c14	*t*	2.2 ± 1.9	11.6	*t*	0.6	2.0	3.4	5.0
19:1 c10	*t*	0.5 ± 1.1	8.2	*t*	*T*	*t*	*t*	1.9
20:1 c5	*t*	4.8 ± 3.1	26.9	2.2	3.1	4.2	5.6	7.6
20:1 c8	*t*	1.3 ± 1.8	10.5	*t*	*T*	0.4	2.1	4.1
20:1 c11 (Gondoic acid)	*t*	8.2 ± 4.9	29.5	2.2	4.8	8.0	11.1	14.1
22:1 c13 (Erucic acid)	*t*	3.9 ± 5.9	48.0	*t*	*T*	*t*	7.1	12.2
24:1 c15 (Nervonic acid)	*t*	4.0 ± 5.3	30.0	*t*	*T*	1.8	6.3	11.8
								
16:1 t9 (Palmitoleic acid)	*t*	17.0 ± 9.1	65.2	*t*	12.1	17.3	22.0	28.2
18:1 t4	*t*	5.2 ± 5.3	30.7	*t*	*T*	4.1	8.3	12.3
18:1 t5 (Thalictric acid)	*t*	1.7 ± 2.9	19.0	*t*	*T*	0.5	1.7	5.6
18:1 t6–8 (Petroselaidic acid)	*t*	7.5 ±5.9	56.0	2.5	3.8	5.7	9.5	14.8
18:1 t9 (Elaidic acid)	*t*	16.5 ± 11.3	88.0	6.4	9.0	13.1	20.0	32.9
18:1 t10	*t*	17.0 ± 11.3	71.1	6.4	8.8	13.7	21.8	32.4
18:1 t11 (Transvaccenic acid)	*t*	14.0 ± 8.1	74.2	5.9	8.7	12.3	18.0	24.7
18:1 t12	*t*	9.6 ± 5.7	42.8	4.0	5.6	8.4	12.3	17.1
18:1 t13 or c6	*t*	12.5 ± 17.1	175.7	*t*	5.7	9.3	13.9	21.1
18:2 tt	*t*	3.5 ± 4.3	38.7	*t*	1.1	2.3	4.5	7.4
18:2 t9t12 (Linoelaidic acid)	*t*	2.1 ± 3.0	20.9	*t*	*T*	1.2	2.5	6.7
18:2 c9t13	*t*	8.5 ± 8.0	69.3	*t*	2.1	7.7	11.9	17.7
18:2 c9t12	*t*	15.4 ± 6.4	45.2	8.4	10.9	14.2	18.9	24.0
18:2 t9c12	*t*	9.8 ± 4.3	31.5	5.3	6.9	9.0	12.2	15.6
								
18:2 c9c14	*t*	3.0 ± 7.7	62.1	*t*	*t*	*t*	*t*	10.0
18:2 c9c15 (Mangiferic acid)	*t*	3.0 ± 3.9	39.1	*t*	*t*	2.0	4.6	7.2
18:2 c9t11 CLA	*t*	14.4 ± 6.2	42.7	7.5	10.1	13.3	17.3	22.4
18:2 c11t13 CLA	*t*	2.1 ± 1.8	12.3	*t*	*t*	2.2	3.0	4.2
18:2 t10c12 CLA	*t*	4.3 ± 2.5	17.9	2.0	2.9	3.8	5.2	7.3
18:2 c/c CLA1	*t*	0.8 ± 1.3	8.5	*t*	*t*	*t*	1.4	2.6
18:2 c/c CLA2	*t*	0.5 ± 1.0	6.6	*t*	*t*	*t*	*t*	1.9
18:2 tt CLA	*t*	6.5 ± 4.4	23.2	*t*	3.2	6.5	9.5	11.9
								
18:2 c9c12 (LA)	279.7	2233.8± 622.6	4970.5	1540.1	1853.0	2208.2	2596.0	2962.0
18:3 c6c9c12 (γ-linolenic acid)	1.4	23.5 ± 13.8	93.3	9.0	13.8	20.8	29.4	41.4
20:2 c11c14 (Dihomo linolenic acid)	*t*	13.1 ± 5.0	37.3	7.8	9.8	12.5	16.0	19.7
20:3 c8c11c14 (Homo-γ-linolenic acid)	7.9	74.3 ± 30.4	222.1	41.5	53.0	68.2	90.4	113.4
20:4 c5c8c11c14 (AA)	42.7	393.0 ± 119.1	882.8	254.8	313.3	385.6	461.4	548.2
22:2 c13c16 (Docosadienoic acid)	*t*	3.1 ± 3.3	18.4	*t*	*t*	2.8	4.9	7.3
22:4 c7c10c13c16 (Adrenic acid)	*t*	15.4 ± 23.0	158.4	1.9	5.6	8.5	12.5	36.5
22:5 c4c7c10c13c16 (n-6 DPA)	*t*	8.0 ± 5.9	41.1	*t*	4.7	7.4	10.3	15.1
18:3 c9c12c15 (LNA)	12.0	54.4 ± 25.1	186.9	29.1	37.9	48.6	65.4	87.6
18:4 c6c9c12c15 (Stearidonic acid)	*t*	0.2 ± 0.5	4.3	*t*	*t*	*t*	*t*	0.6
20:3 c11c14c17 (Dihomolinoleic acid)	*t*	1.6 ± 2.5	17.9	*t*	*t*	*t*	2.9	4.8
20:5 c5c8c11c14c17 (EPA)	4.4	40.3 ± 28.3	215.4	16.0	23.4	32.4	47.5	73.3
22:3 c13c16c19	*t*	0.6 ± 1.9	15.1	*t*	*t*	*t*	*t*	2.6
22:5 c7c10c13c16c19 (n-3 DPA)	*t*	23.9 ± 10.0	88.5	14.0	17.7	22.1	27.8	36.5
22:6 c4c7c10c13c16c19 (DHA)	7.2	88.8 ± 36.8	237.5	47.8	62.7	82.0	110.6	138.0
Total FA	1251.1	6947.6±1816.2	16225.3	5052.5	5780.4	6745.4	7947.8	9108.9

Abbreviation: FA, fatty acids; *t*, trace. N = 826.

**Table 4:** Studies reporting circulating FA levels (Concentration *vs*. % composition) in the past 5 years.

Study	# Subjects	Age range	# FA^‡^	% / Conc	Health status
*Articles reporting FA concentrations*					
Klein CJ et al. 2013; 28:87–94.[38]	10	˂ 1	10	Conc	Hypebilirubineamia
Sauerwald UC et al. 2012 Mar;54(3):353–63 [39].	66	˂ 1	12	Conc	Preterm
El-Ansary AK et al. 2011; 10:62 [40].	52	4–12	4	Conc	Normal and autistic
Neggers YH et al. 2009;18(1):22–8 [41].	62	˂ 13	19	Conc	Healthy & mental retardation
Mehmetoqlu I et al. 2012;21(4):519–25.	161	21–60	*na* ^$^	Conc	Normal & severe obesity
Khaw KT et al. 2012; 9(7): e1001255[42].	10000	40–79	22	Conc	Healthy and CHD
Cunnane SC et al. 2012; 29(3):691–7[43].	36	*ns*	8	Conc	Cognitive impairment and Alzheimer’s disease
					
*Articles reporting % composition*					
Meldrum SJ et al. 2012 Jun;86(6):233–9 [44].	420	˂ 1	1	%	Healthy
Miller MR et al. 2010 Oct;6(4):338–46 [45].	110	˂ 1	2	%	Healthy
Sabel KG et al. 2009 Jun 10;8:20 [46].	91	Infants, >40	4	%	Healthy
Chien et al. 2011; 10:33[47].	1986	5	2	%	Healthy & Met S
Steer CD et al. 2012 Apr 1;21(7):1504–12 [48].	5632	7	16	%	Healthy
Zhou YE et al. 2009;58(2):158–66 [49].	178	12–16	14	%	Healthy
Bokor S et al. 2010 Aug;51(8):2325–33 [50].	1144	13–16	4	%	Healthy
Gallo S et al. 2010 May;95(5):2410–7 [51].	180	13–17	6	%	Healthy
Wheeler SJ et al. 2011;105(4):601–10 [52].	283	14–18	16	%	Healthy pregnant
Bradbury KE et al. 2011; 3: 152–163 [18].	2793	15->65	13	%	Healthy
Garneau V et al. 2012 Jul 9;11:46 [20].	198	18–55	4	%	Healthy
Garneau V et al. 2013; 38(3):243–8 [20].	100	18–55	3	%	Healthy
Ottestad I et al. 2012 Jul;108(2):315–26 [53].	54	18–50	6	%	Healthy
Glew RH et al. 2010; 28 (2): 159–166 [54].	51	>18	26	%	Healthy
Chorell E et al. 2012 Apr;8(4):1187–96 [55].	29	19–33	8	%	Healthy
Telle-Hansen VH et al. 2012 Feb;47(2):151–60 [56].	38	20–40	7	%	Healthy
Schuchardt JP et al. 2011 Aug 22;10:145 [57].	12	20–50	2	%	Healthy
Mathias RA et al. 2011 May 20;12:50 [58].	155	25–50	4	%	MetS
Buydens-Branchey L et al. 2011 Aug 15;188(3):422–7. [59]	25	30–45	6	%	Cocaine Abuse
Kim JY et al. 2010 Sep 3;9(9):4368–75 [60].	60	30–50	13	%	Lean and overweight/obese
Park Y et al. 2009 Aug;12(4):803–8 [61].	136	30–60	*na* ^$^	%	Hypertriglyceridemia
Tanaka T et al. 2009 Jan; 5(1): e1000338 [24].	2151	30–85	6	%	Healthy
Kawashima A et al. 2009;55(5):400–6 [62].	94	35–70	11	%	MetS and abdominal obesity
Perez-Martinez P et al. 2012 Feb;56(2):309–15 [63].	452	35–70	*na* ^$^	%	MetS
Lee S et al. 2012 Feb;107(4):567–72 [64].	926	40–49	3	%	Healthy
Woods MN et al. 2009 Apr;89(4):1180–7 [65].	70	40–55	15	%	HIV & hypertriglyceridemia
Rasic-Milutinovic Z et al. 2012 Jan;43(1):75–82 [66].	36	40–65	11	%	Healthy & Type2 diabetes
Kwak JH et al. 2011 Jan;214(1):94–100 [67].	1646	40–79	10	%	Healthy and CAD
Steffen BT et al. 2012 Jun;36(6):797–804 [68].	2848	45–48	3	%	Healthy
Wilk JB et al. 2012; 96: 882–8 [6].	1575	45–65	1	%	Healthy
Park Y et al. 2009 Dec;29(12):825–30 [69].	68	45–65	16	%	Ischemia and hemorrhagic stroke
Sergeant S et al. 2012 Feb;107(4):547–55 [70].	166	50–75	4	%	Healthy & Type 2 diabetes
Tan ZS et al. 2012;78(9); 658–64 [71].	1575	55–70	2	%	Healthy
Zulyniak MA et al. 2012 Oct;37(5):1003–7 [72].	20	*ns*	14	%	Healthy & HI & HG
Holub BJ et al. 2009 Dec 24;8:58 [73].	2053	*ns*	1	%	Healthy

Abbreviations: FA: fatty acids; Conc: concentration; CHD: coronary heart disease; MetS: metabolic syndrome; CAD: coronary artery disease; HG: hyperglycemia; HI: hyperinsulinemia; *ns*: not specified; *na*: not applicable.

^ǂ^ # of FA does not include sums of FA groups but only individual FA of which levels have been reported.

^$^Articles reported sums of FA groups but not levels of individual FA.

In the present study concentrations of circulating FA were compared by sex and ethnicity (Tables 5 and 6, respectively). From the 61 FA investigated five were significantly different between males and females: palmitoleic acid, linoleic acid (LA) and γ-linolenic acid (GLA) and docosapentaenoic acid (n-3 DPA) and docosahexaenoic acid (DHA) (Table 5). Our findings show that males had significantly higher GLA and DPA concentrations than females (p ˂ 0.05) while females had significantly higher palmitoleic acid, LA and DHA concentrations than males (p < 0.05). Previously, in a self-selected dietary intake study, in 29 healthy adults from the U.S aged 20–59 years old, higher plasma LA concentrations were reported in females compared to males [19]. To the best of our knowledge no other studies have reported increased GLA concentrations in males compared to females. The significantly increased DPA concentrations in males observed in this study was also reported previously in a study of 200 subjects, from Quebec city, aged 18–55 years old [20]; however, DPA levels were measured in the plasma phospholipid fraction, not total lipids. Increased levels of DHA and decreased levels in DPA in females compared to males have been attributed to higher rate of DHA synthesis in females [21,22]. Giltay et al have attributed higher DHA concentrations in females to estrogen [23]. The differences observed in FA concentrations between sexes in this study may also be attributed to differences in hormones or genetic variations [24].

**Table 5:** Concentration (μmol/L) of select FA in males and females.

FA	Males	Females	p- value
	n = 327	n = 499	
16:0	1648.6 ± 487.9	1620.0 ± 440.5	0.37
18:0	483.8 ± 121.9	492.9 ± 126.0	0.30
16:1 c9	129.7 ± 67.8	135.2 ± 66.8	0.01*
18:1 c9	1332.7 ± 454.5	1275.0± 390.8	0.28
18:1 c11	131.2 ± 61.0	127.8 ± 58.6	0.41
18:2 c9c12	2174.6 ± 599.0	2272.06 ± 634.5	0.03*
18:3 c6c9c12	26.0 ± 15.1	21.9 ± 12.7	0.01*
18:3 c9c12c15	55.1 ± 27.5	53.9 ± 23.5	0.86
20:3 c8c11c14	78.4 ± 32.1	71.6 ± 29.0	0.19
20:4 c5c8c11c14	403.9 ± 125.1	385.7 ± 114.9	0.27
20:5 c5c8c11c14c17	39.5 ± 25.1	40.6 ± 30.1	0.27
22:5 c7c10c13c16c19	25.3 ± 11.1	23.0 ± 9.1	˂ 0.01*
22:6 c4c7c10c13c16c19	81.0 ± 31.8	93.6 ± 38.8	˂ 0.01*

Data represented as Mean±SD.

*A p-value < 0.05 was considered statistically significant.

Linear regression models were adjusted for age, BMI, physical activity, % Energy from dietary fat, and ethnicity. Abbreviation: FA, fatty acids.

**Table 6:** Concentration (μmol/L) of select FA in Caucasians, East Asians and South Asians.

FA	Caucasians	East Asians	South Asians	p- value
	n = 287	n = 353	n = 107	
16:0	1654.5 ± 527.1	1646 ± 420.7	1558.1 ± 426.5	0.13
18:0	488.1 ± 136.1	498.1 ± 123.6	473.7 ± 105.3	0.62
16:1 c9	142.7 ± 79.5^a^	133.0 ± 59.6^a^	113.1 ± 56.6^b^	˂ 0.01*
18:1 c9	1322.5 ± 466.1^a^	1288.1 ± 399.4^a, b^	1205.5 ± 393.2^b^	0.01*
18:1 c11	131.1 ± 66.0	132.0 ± 53.1	117.6 ± 59.1	0.18
18:2 c9c12	2144.7 ± 638.8	2352.0 ± 630.0	2145.0 ± 543.3	0.34
18:3 c6c9c12	25.9 ± 13.9^a^	19.9 ± 13.7^b^	28.2 ± 13.3^a^	0.02*
18:3 c9c12c15	51.9 ± 25.2	56.5 ± 25.2	57.7 ± 27.1	0.20
20:3 c8c11c14	82.4 ± 34.7^a^	65.9 ± 25.5^b^	76.0 ± 27.8^a^	0.03*
20:4 c5c8c11c14	401.8 ± 129.4	376.6 ± 108.4	412.6 ± 111.6	0.25
20:5 c5c8c11c14c17	39.2 ± 27.0	43.2 ± 31.8	36.1 ± 18.6	0.55
22:5 c7c10c13c16c19	24.5 ± 11.5	23.6 ± 8.9	23.9 ± 10.1	0.72
22:6 c4c7c10c13c16c19	78.2 ± 35.1	103.6 ± 36.3	73.9 ± 31.0	0.79

Data represented as Mean±SD. Different letters (^a/b^) denote values that are significantly different between groups.

*A p-value < 0.05 was considered statistically significant.

Linear regression models were adjusted for age, BMI, physical activity, energy intake from fat, and sex. Abbreviation: FA, fatty acids.

Comparison of FA concentrations between Caucasians, East Asians and South Asians revealed that South Asians had significantly lower concentrations of palmitoleic acid and oleic acid while East Asians had lower concentrations of GLA and dihomo-γ-linolenic (DGLA) acid (Table 6). The low levels of GLA and DGLA in East Asians are consistent with findings from the Multi-Ethnic Study of Atherosclerosis [25]. Differences in circulating FA concentrations between ethnicities can be a result of genetic variations [14].

Differences in circulating FA concentrations identified in this study give insight into sex- and ethnicity-specific susceptibility to health outcomes. Studies have revealed significant associations between various circulating FA and risk of chronic disease. For instance plasma levels of GLA and DGLA have been shown to be positively associated, whereas levels of LA, EPA and DHA are inversely associated, with biomarkers of inflammation [25,26]. Plasma levels of DGLA are also associated with depression and insulin resistance [27,28]. Levels of palmitoleic acid are positively associated, while levels of LA are inversely associated, with ischemic stroke and insulin resistance [28,29]. On the other hand the reason for the strikingly small number of FA that are different between sexes or ethnicities could be attributed to diet. Although our study cohort consisted of ethnically diverse males and females, participants were young Canadians and many were students that were more likely to share similar dietary habits. We also acknowledge that although this is a randomly sampled free living population, the dietary habits and FA profile of participants in this study, all being from Toronto, cannot be generalized to other regions of Canada and the world. The young age and healthy status of our study participants may also explain the weak positive correlations, albeit significant, between FA and LDL, triglycerides and total cholesterol reported in Tables 7, 8, and 9, respectively. Correlations between FA and BMI, HOMA-IR, glucose, insulin, HDL, free fatty acids were also investigated; however, R^2^ values equal or higher than 0.09 were not found (data not shown). The weak correlations are reflective of the healthy status of our study cohort. Nonetheless, the correlations shown exemplify the potential use of FA as biomarkers of health. As such, future studies will include participants with wider age range to capture metabolic changes in aging populations. Determining concentrations of FA in aging and unhealthy individuals will allow for the identification of correlations of FA with biomarkers of health, which will aid in establishing FA reference ranges.

**Table 7:** Correlation between select plasma FA and LDL-cholesterol.

FA	R^2^	p- value
14:0	0.09	˂ 0.0001*
15:0	0.12	˂ 0.0001*
16:0	0.20	˂ 0.0001*
16:1 c9	0.09	˂ 0.0001*
18:0	0.18	˂ 0.0001*
18:1 c9	0.15	˂ 0.0001*
18:1 c11	0.09	˂ 0.0001*
18:2 c9c12	0.20	˂ 0.0001*
18:3 c9c12c15	0.10	˂ 0.0001*
20:2 c11c14	0.09	˂ 0.0001*
20:3 c8c11c14	0.14	˂ 0.0001*
20:4 c5c8c11c14	0.19	˂ 0.0001*
22:5 c7c10c13c16c19	0.12	˂ 0.0001*
22:6 c4c7c10c13c16c19	0.09	˂ 0.0001*

Linear regression models were adjusted for age, BMI, physical activity, energy intake from fat, sex and ethnicity. All correlations identified below are positive. R^2^ corresponds to the coefficient of determinations. Only R^2^ with a value of 0.09 or more were reported.

*A p-value < 0.05 was considered statistically significant. N = 826. Abbreviation: FA, fatty acids.

**Table 8:** Correlations between select plasma FA and triglycerides.

FA	R^2^	p- value
14:0	0.36	˂ 0.0001*
14:1	0.15	˂ 0.0001*
15:0	0.16	˂ 0.0001*
16:0	0.31	˂ 0.0001*
16:1 t9	0.14	˂ 0.0001*
16:1 c9	0.29	˂ 0.0001*
18:0	0.14	˂ 0.0001*
18:1 t11	0.09	˂ 0.0001*
18:1 c9	0.40	˂ 0.0001*
18:2 c9t12	0.11	˂ 0.0001*
18:2 c9c12	0.10	˂ 0.0001*
18:3 c6c9c12	0.17	˂ 0.0001*
20:1 c11	0.11	˂ 0.0001*
18:3 c9c12c15	0.26	˂ 0.0001*
18:2 c9t11 CLA	0.24	˂ 0.0001*
21:0	0.10	˂ 0.0001*
20:2 c11c14	0.13	˂ 0.0001*
20:3 c8c11c14	0.16	˂ 0.0001*
22:2 c13c16	0.14	˂ 0.0001*
22:5 c7c10c13c16c19	0.10	˂ 0.0001*

Linear regression models were adjusted for age, BMI, physical activity, energy intake from fat, sex and ethnicity. All correlations identified are positive. R^2^ corresponds to the coefficient of determinations. Only R^2^ with a value of 0.09 or more were reported.

*A p-value < 0.05 was considered statistically significant. N = 826. Abbreviation: FA, fatty acids.

**Table 9:** Correlations between select plasma FA and total cholesterol.

FA	R^2^	p- value
14:0	0.14	˂ 0.0001*
15:0	0.14	˂ 0.0001*
16:0	0.26	˂ 0.0001*
16:1 t9	0.09	˂ 0.0001*
16:1 c9	0.15	˂ 0.0001*
18:0	0.25	˂ 0.0001*
18:1 c9	0.20	˂ 0.0001*
18:2 c9c12	0.29	˂ 0.0001*
18:3 c6c9c12	0.09	˂ 0.0001*
18:3 c9c12c15	0.11	˂ 0.0001*
18:2 c9t11 CLA	0.10	˂ 0.0001*
20:2 c11c14	0.12	˂ 0.0001*
20:3 c8c11c14	0.15	˂ 0.0001*
20:4 c5c8c11c14	0.18	˂ 0.0001*
22:2 c13c16	0.09	˂ 0.0001*
22:5 c7c10c13c16c19	0.13	˂ 0.0001*
22:6 c4c7c10c13c16c19	0.14	˂ 0.0001*

Linear regression models were adjusted for age, BMI, physical activity, energy intake from fat, sex and ethnicity. All correlations identified were positive. R^2^ corresponds to the coefficient of determinations. Only R^2^ with a value of 0.09 or more were reported.

*A p-value < 0.05 was considered statistically significant. N = 826. Abbreviation: FA, fatty acids.

Generally, epidemiological studies investigating the link between FA intake and disease often depend on food frequency questionnaires (FFQ) for the estimation of exposure to different types of FA. The limitations of detailed dietary intake records are well documented and these include dependence on participants recall and bias [30]. In addition FFQ do not reflect the inter-individual differences in metabolism, absorption and genetic variations leading to different concentrations of circulating FA. Correlation studies between food intake and circulating FA levels in US women revealed that circulating levels of saturated and monounsaturated FA did not reflect intake, possibly as a result of endogenous FA synthesis [31]. Taken together exposure to FA should be determined objectively by measuring blood or tissue levels of FA as opposed to dietary levels. Measurement of plasma as an aggregate of both dietary and de novo synthesis of FA may be more appropriate for assessing linkages to biomarkers. A relevant example is the measurement of blood cholesterol, the net contribution of dietary and de novo synthesis, for ascertaining cardiovascular disease risk.

FA are commonly measured in adipose tissue, erythrocytes or plasma. In this study FA concentrations were determined in plasma total lipids. While FA levels in adipose tissue reflect intake in years [32] and levels in erythrocytes reflect intake in months [31], FA levels in plasma reflect intake in weeks [33] and; therefore, are more reflective of current dietary habits of subjects. Availability of adipose tissue limits its use in epidemiological studies and similarly excludes its appropriateness for rapid and frequent determination of endogenous FA levels [33]. We recognize that both plasma and red blood cells are commonly used for assessing circulating levels of fatty acids. A recent report by Skeaff et al have challenged the notion that plasma only reflects current intake by showing that plasma fatty acids levels correlates with intake for up to 2 weeks [34]. Studies have also shown that red blood cell fatty acid levels do not correlate with dietary saturated and monounsaturated fatty acids because of the contribution of *de novo* synthesis to their circulating levels. The study by Patel et al showed stronger associations between disease risk and plasma FA compared to erythrocyte fatty acids [35]. Measuring FA levels in total plasma lipids is more applicable to large populations due to simplicity of the analytical methodology [36]. Furthermore, plasma is composed of all major circulating lipid species including triglycerides, phospholipids, cholesterol-esters and free fatty acids [37]. Therefore, plasma provides a highly accessible source of lipids and provides a high level overview of changes in metabolism of all these lipid species as a potential indicator of health.

In conclusion the present study provides knowledge regarding a broad panel of circulating FA. The generalizability of this study requires further replication in other populations but these data are the first step in establishing FA reference ranges which is a vital gap in elucidating the role of individual FA in chronic disease. Further research is warranted, building upon the present results, to examine how very high or low circulating FA concentrations relate to different chronic diseases.

## Supporting Information

S1 Table Study raw data containing subjects’ fatty acids concentrations and other characteristics. (XLSX)
