# Correction: Comprehensive Profiling of Plasma Fatty Acid Concentrations in Young Healthy Canadian Adults

**DOI:** 10.1371/journal.pone.0128167

**Published:** 2015-05-13

**Authors:** The PLOS ONE Staff

There are a number of errors in Table 3. Multiple upper-case T’s were included instead of the correct lower-case, italicized *t*’s. Trans-vaccenic acid was incorrectly spelled as Transvaccenic acid, and Palmitelaidic acid was incorrectly written as Palmitoleic acid. Please see the correct Table 3 here.

**Table 3:** Range, mean and percentiles of FA concentrations (μmol/L) of plasma total lipids.

Fatty Acid	Min	Mean±SD	Max	10	25	50	75	90
14:0 (Myristic acid)	16.2	63.6 ± 37.1	325.7	29.8	39.2	54.0	76.2	104.7
15:0 (Pentadyclic acid)	*t*	17.8 ± 6.7	56.1	10.8	13.2	17.1	21.3	26.3
16:0 (Palmitic acid)	285.4	1631.1 ± 459.3	4064.5	1140.2	1339.7	1569.5	1839.2	2182.2
18:0 (Stearic acid)	110.2	489.5 ± 124.3	1013.7	353.3	406.9	474.2	556.8	650.9
19:0	*t*	4.3 ± 4.2	25.7	*t*	1.1	3.1	6.6	10.2
20:0 (Arachidic acid)	*t*	4.9 ± 3.7	29.8	*t*	2.6	4.6	6.4	9.4
21:0	*t*	1.5 ± 1.9	10.0	*t*	*t*	0.3	2.9	4.1
22:0 (Behenic acid)	*t*	6.7 ± 5.3	39.0	*t*	3.4	6.0	9.4	14.0
24:0 (Lignoceric acid)	*t*	1.4 ± 2.4	15.7	*t*	*t*	*t*	2.4	5.1
								
14:1 (Myristoleic acid)	*t*	2.7 ± 4.1	31.3	*t*	*t*	*t*	4.2	7.7
15:1 c10	*t*	0.1 ± 0.3	2.7	*t*	*t*	*t*	*t*	*t*
16:1 c9 (Palmitoleic acid)	27.7	133.0 ± 67.2	555.9	67.8	88.5	119.5	156.9	211.4
17:1 c10	*t*	10.5 ± 7.4	45.2	*t*	6.2	10.7	14.7	19.1
18:1 c9 (Oleic acid)	178.7	1285.5± 416.7	3210.5	858.6	1007.1	1226.3	1472.0	1808.7
18:1 c11 (Vaccenic acid)	11.4	129.2 ± 59.5	562.9	81.5	96.6	114.3	141.5	185.1
18:1 c12	*t*	18.7 ± 13.6	101.8	6.6	9.0	14.9	22.7	38.3
18:1 c13	*t*	3.5 ± 3.5	18.1	*t*	1.2	2.5	4.5	8.9
18:1 c14	*t*	2.2 ± 1.9	11.6	*t*	0.6	2.0	3.4	5.0
19:1 c10	*t*	0.5 ± 1.1	8.2	*t*	*t*	*t*	*t*	1.9
20:1 c5	*t*	4.8 ± 3.1	26.9	2.2	3.1	4.2	5.6	7.6
20:1 c8	*t*	1.3 ± 1.8	10.5	*t*	*t*	0.4	2.1	4.1
20:1 c11 (Gondoic acid)	*t*	8.2 ± 4.9	29.5	2.2	4.8	8.0	11.1	14.1
22:1 c13 (Erucic acid)	*t*	3.9 ± 5.9	48.0	*t*	*t*	*t*	7.1	12.2
24:1 c15 (Nervonic acid)	*t*	4.0 ± 5.3	30.0	*t*	*t*	1.8	6.3	11.8
								
16:1 t9 (Palmitelaidic acid)	*t*	17.0 ± 9.1	65.2	*t*	12.1	17.3	22.0	28.2
18:1 t4	*t*	5.2 ± 5.3	30.7	*t*	*t*	4.1	8.3	12.3
18:1 t5 (Thalictric acid)	*t*	1.7 ± 2.9	19.0	*t*	*t*	0.5	1.7	5.6
18:1 t6-8 (Petroselaidic acid)	*t*	7.5 ±5.9	56.0	2.5	3.8	5.7	9.5	14.8
18:1 t9 (Elaidic acid)	*t*	16.5 ± 11.3	88.0	6.4	9.0	13.1	20.0	32.9
18:1 t10	*t*	17.0 ± 11.3	71.1	6.4	8.8	13.7	21.8	32.4
18:1 t11 (Trans-vaccenic acid)	*t*	14.0 ± 8.1	74.2	5.9	8.7	12.3	18.0	24.7
18:1 t12	*t*	9.6 ± 5.7	42.8	4.0	5.6	8.4	12.3	17.1
18:1 t13 or c6	*t*	12.5 ± 17.1	175.7	*t*	5.7	9.3	13.9	21.1
18:2 tt	*t*	3.5 ± 4.3	38.7	*t*	1.1	2.3	4.5	7.4
18:2 t9t12 (Linoelaidic acid)	*t*	2.1 ± 3.0	20.9	*t*	*t*	1.2	2.5	6.7
18:2 c9t13	*t*	8.5 ± 8.0	69.3	*t*	2.1	7.7	11.9	17.7
18:2 c9t12	*t*	15.4 ± 6.4	45.2	8.4	10.9	14.2	18.9	24.0
18:2 t9c12	*t*	9.8 ± 4.3	31.5	5.3	6.9	9.0	12.2	15.6
								
18:2 c9c14	*t*	3.0 ± 7.7	62.1	*t*	*t*	*t*	*t*	10.0
18:2 c9c15 (Mangiferic acid)	*t*	3.0 ± 3.9	39.1	*t*	*t*	2.0	4.6	7.2
18:2 c9t11 CLA	*t*	14.4 ± 6.2	42.7	7.5	10.1	13.3	17.3	22.4
18:2 c11t13 CLA	*t*	2.1 ± 1.8	12.3	*t*	*t*	2.2	3.0	4.2
18:2 t10c12 CLA	*t*	4.3 ± 2.5	17.9	2.0	2.9	3.8	5.2	7.3
18:2 c/c CLA1	*t*	0.8 ± 1.3	8.5	*t*	*t*	*t*	1.4	2.6
18:2 c/c CLA2	*t*	0.5 ± 1.0	6.6	*t*	*t*	*t*	*t*	1.9
18:2 tt CLA	*t*	6.5 ± 4.4	23.2	*t*	3.2	6.5	9.5	11.9
								
18:2 c9c12 (LA)	279.7	2233.8± 622.6	4970.5	1540.1	1853.0	2208.2	2596.0	2962.0
18:3 c6c9c12 (γ-linolenic acid)	1.4	23.5 ± 13.8	93.3	9.0	13.8	20.8	29.4	41.4
20:2 c11c14 (Dihomo linolenic acid)	*t*	13.1 ± 5.0	37.3	7.8	9.8	12.5	16.0	19.7
20:3 c8c11c14 (Homo-γ-linolenic acid)	7.9	74.3 ± 30.4	222.1	41.5	53.0	68.2	90.4	113.4
20:4 c5c8c11c14 (AA)	42.7	393.0 ± 119.1	882.8	254.8	313.3	385.6	461.4	548.2
22:2 c13c16 (Docosadienoic acid)	*t*	3.1 ± 3.3	18.4	*t*	*t*	2.8	4.9	7.3
22:4 c7c10c13c16 (Adrenic acid)	*t*	15.4 ± 23.0	158.4	1.9	5.6	8.5	12.5	36.5
22:5 c4c7c10c13c16 (n-6 DPA)	*t*	8.0 ± 5.9	41.1	*t*	4.7	7.4	10.3	15.1
18:3 c9c12c15 (LNA)	12.0	54.4 ± 25.1	186.9	29.1	37.9	48.6	65.4	87.6
18:4 c6c9c12c15 (Stearidonic acid)	*t*	0.2 ± 0.5	4.3	*t*	*t*	*t*	*t*	0.6
20:3 c11c14c17 (Dihomolinoleic acid)	*t*	1.6 ± 2.5	17.9	*t*	*t*	*t*	2.9	4.8
20:5 c5c8c11c14c17 (EPA)	4.4	40.3 ± 28.3	215.4	16.0	23.4	32.4	47.5	73.3
22:3 c13c16c19	*t*	0.6 ± 1.9	15.1	*t*	*t*	*t*	*t*	2.6
22:5 c7c10c13c16c19 (n-3 DPA)	*t*	23.9 ± 10.0	88.5	14.0	17.7	22.1	27.8	36.5
22:6 c4c7c10c13c16c19 (DHA)	7.2	88.8 ± 36.8	237.5	47.8	62.7	82.0	110.6	138.0
Total FA	1251.1	6947.6±1816.2	16225.3	5052.5	5780.4	6745.4	7947.8	9108.9

QALY: Quality-adjusted life years; CHD: Coronary heart disease; CKD: Chronic kidney disease; OI: Opportunistic infection; AE: Adverse event; VL: Viral load.

## References

[1] Abdelmagid SA, Clarke SE, Nielsen DE, Badawi A, El-Sohemy A, Mutch DM (2015) Comprehensive Profiling of Plasma Fatty Acid Concentrations in Young Healthy Canadian Adults. PLoS ONE 10(2): e0116195 doi:10.1371/journal.pone.0116195 25675440 10.1371/journal.pone.0116195 PMC4326172

