# Correction: Comprehensive Profiling of Plasma Fatty Acid Concentrations in Young Healthy Canadian Adults

**DOI:** 10.1371/journal.pone.0315115

**Published:** 2024-12-03

**Authors:** Salma A. Abdelmagid, Shannon E. Clarke, Daiva E. Nielsen, Alaa Badawi, Ahmed El-Sohemy, David M. Mutch, David W. L. Ma

In Fig 1, the image in panels A and B are switched and the x-axis are labelled incorrectly. Please see the correct Fig 1 here.

**Fig 1:**
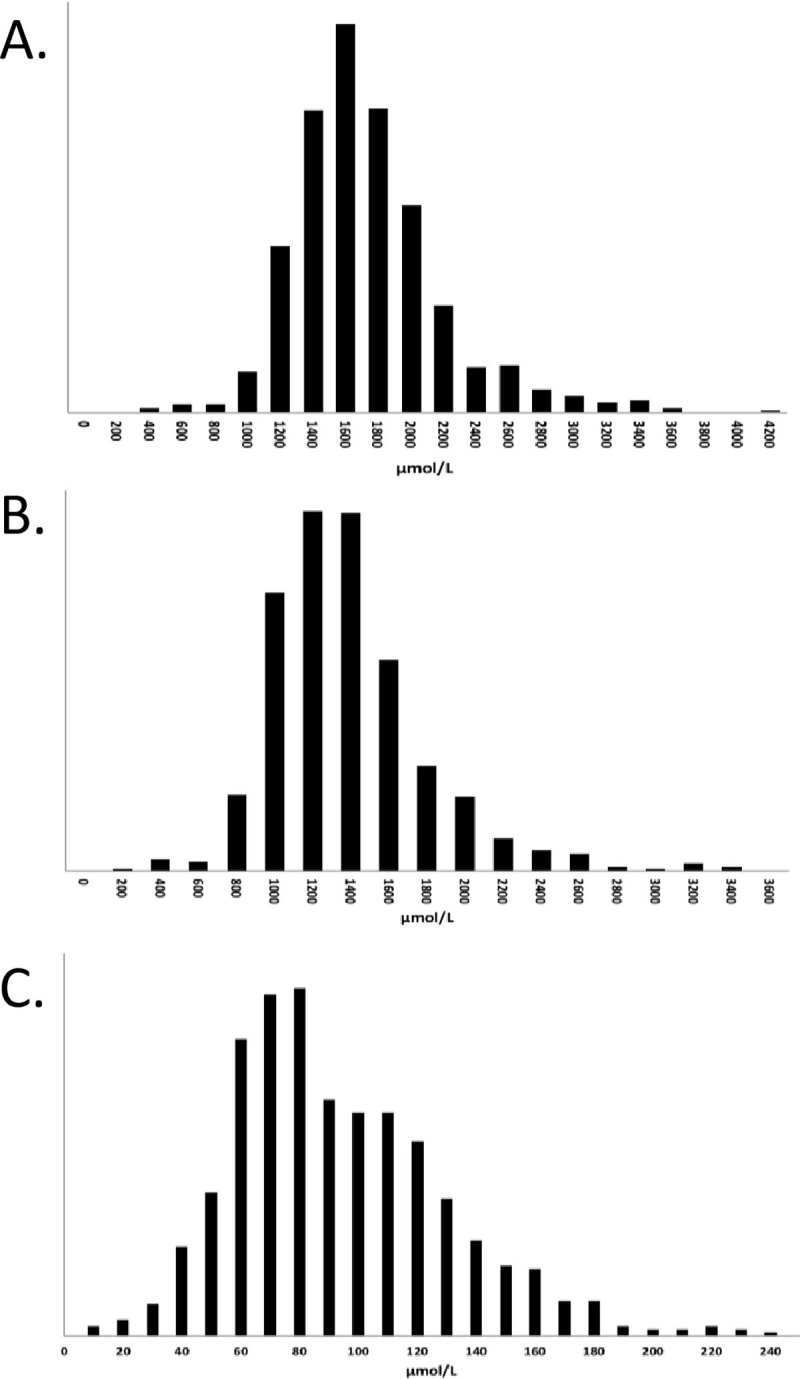
Distribution of total plasma fatty acid concentrations of selected fatty acids. A. 16:0 (Palmitic acid); B. 18:1 c9 (Oleic acid); C. 22:6n-3 (DHA).

## References

[1] Abdelmagid SA, Clarke SE, Nielsen DE, Badawi A, El-Sohemy A, Mutch DM, et al. (2015) Comprehensive Profiling of Plasma Fatty Acid Concentrations in Young Healthy Canadian Adults. PLoS ONE 10(2): e0116195. doi: 10.1371/journal.pone.0116195 25675440 PMC4326172

